# Statistics, truth finding and predictions: what every gynaecologist and researcher should know

**DOI:** 10.52054/FVVO.15.2.080

**Published:** 2023-06-30

**Authors:** P.R. Koninckx, A Wattiez, E Saridogan

**Affiliations:** Prof emeritus Obstetrics and Gynecology KULeuven Belgium, the University of Oxford UK, University Cattolica, Rome, Italy and Moscow State University; Latifa Hospital, UAE ; Prof university of Strasbourg, Strasbourg, France; Elizabeth Garrett Anderson Institute for Women’s Health, University College London, United Kingdom

## You do not have to understand a motor to drive a car

### Population, samples and distributions

**Figure 1:**
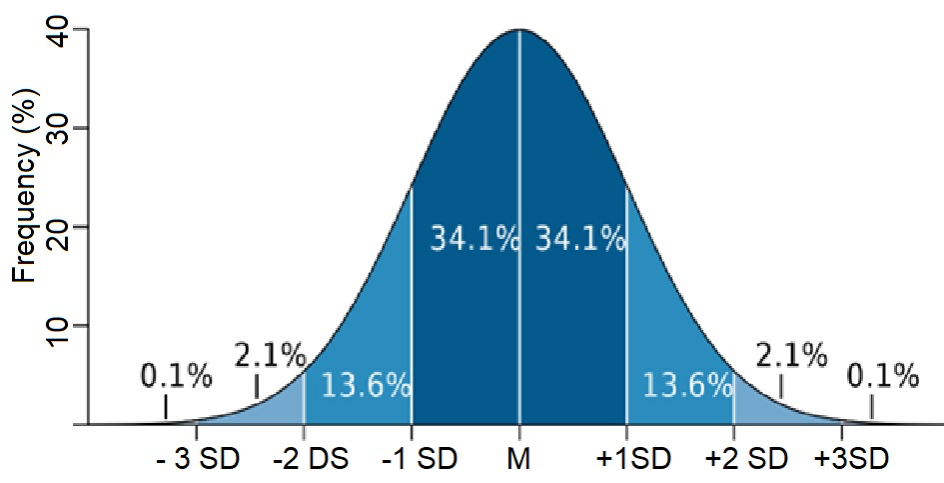
Frequency distribution of a normal distribution and the percentage of the population in the mean plus and minus, 1, 2, or 3 SDs, respectively.

Medicine and research started with observing events and learning from them. Most biological observations, such as height and weight, are normally or Gaussian distributed. The frequency distribution of the variability in, e.g. height is a bell-shaped curve with many observations around the mean height and few very tall or small. The beauty of a normal distribution is that it is accurately described by a formula with only two variables, the mean (M) and a standard deviation (SD) and that 68% of all observations are between the M + 1 SD and 95% between M + 2 SDs (Figure 1). Percentiles similarly indicate the distance from the mean, describing the percentage of values smaller and larger. For example, for a value equal to the M + 2 SDs, only 2.2% will be more elevated. The normal distribution also explains that cumulative frequencies are often sigmoidal.

However, the distribution of most biological variables is not linear but logarithmic, which is similar to our common language of saying twice as tall, twice as frequent etc. For example, since blood concentrations of hormones have a logarithmic distribution, a mean concentration of 10 can have as 10th and 90th percentiles, 3 and 30, i.e. three times smaller or larger. The 1st percentile will be a little above zero, i.e. little or no hormone. If this logarithmic, skewed distribution on a linear scale is not considered, the M - 3 SDs will be below zero, which is nonexistent.

To compare the mean of two groups, the accuracy of the estimation of the mean is important. This accuracy, estimated by the standard error of the mean (SEM), improves with the square root of the number of observations.

### Traditional or frequentist statistics and p-values

Traditional statistics (Fisher, 1925) answer with one value, the probability that a difference between two sets of observations, e.g. the height of men and women, can be obtained by chance during sampling. With a null hypothesis of no difference, this probability is the p-value. A p-value of 0.05 indicates a probability of 5% that observed differences could be explained by chance, at least if sampling was done without biases, such as both groups not being from the same country and race. Therefore, testing other hypotheses, such as subgroup analysis, requires considering these hypotheses before sampling. However, subgroup testing and p-values can give valuable indications to be tested in another properly designed trial.

Traditional statistics, thus, can refute a hypothesis when it is likely that results can be explained by chance. However, traditional statistics cannot confirm a hypothesis since the null hypothesis of no difference. This is known as the p-value fallacy (Goodman, 1999), and this mistake is frequently made in medicine, as discussed by the American Statistical Association (Wasserstein and Lazar, 2016). For this and other reasons, most of the conclusions in medical journals were erroneous, as estimated indirectly by a Bayesian approach (Ioannidis, 2005).

A p-value indicating the probability of results being obtained by chance, it is arbitrary to consider 5% as significant and 6% as not. Therefore, it was suggested to interpret and use p-values and abandon the word “significant” (Farland et al., 2016; Wasserstein and Lazar, 2016; Matthews, 2019; Wilson and Falcone, 2020).

The p-value has been criticised from the beginning for two reasons. The magnitude of the effect and the power of the study are not considered. Since the accuracy of the estimation of the mean increases with the square root of the number of observations, tiny, clinically insignificant differences can reach a p-value of 0.05 if the number of observations is large enough. Therefore, confidence intervals should be given. A more fundamental criticism was that results were reduced to one hypothesis of no difference, disregarding alternate hypotheses and previous observations (Neyman and Pearson, 1928).

Unfortunately, evidence-based medicine (EBM) mainly uses p-values and significances, and thus also, guidelines might be biased.

### Predictive and Bayesian statistics

All available data should be used to estimate the probability that a hypothesis is true, not only those of the last experiment. New data should be used to update our knowledge. Although the Bayesian theorem was formulated in 1763, and despite the criticism of the p-value (Neyman and Pearson 1928), the introduction of Bayesian statistics (Lesaffre and Lawson, 2012) in medicine is relatively recent.

The principle of updating knowledge based on prior data with new data is similar to human behaviour and learning from the past. This is similar to updating cooking or managing endometriosis by the results of previous cooking or previous diagnoses and therapies. Learning from the past and updating knowledge is widely used. Examples include the weather forecast, self-driving cars and hedge funds. Bayesian statistics are about estimating the uncertainty of predictions.

The ‘diagnosis AND treatment AND result’ in a patient is a new experiment, and the clinician will use this information to update the management of the next patient. This is the individual experience of the clinician, which in the pyramid of evidence is considered of little value because of the many potential biases.

### Clinical medicine is complex.

Clinical medicine is complex, and most diagnoses, decisions and effects are multifactorial. For example, the decision to do surgery for an ovarian cyst will vary with the woman’s age, degree of pain, size of the cyst, and potential diagnoses. The clinician uses all factors simultaneously, and the evaluation of each factor separately simplifies reality, the relationship between the outcome and one causal factor being a line, two causal factors being an area, and three factors being a sphere. Clinical medicine combines the antecedents of the patient AND symptoms AND clinical exam AND imaging AND blood exams to estimate the probability of each of the several alternative diagnoses from the most to the less likely, and considers the risk of missing a rare but important diagnosis such as cancer.

An inherent weakness of the RCT is that they are not suited for rare events and for evaluating multiple factors and their interaction. Both would require prohibitively large groups or complex randomisation issues.

### Research versus clinical medicine

Research is about understanding mechanisms and pathways of physiology, molecular biology, brain function and treating diseases. Research generally estimates the mean or the variability of variables, with traditional statistics being a powerful tool to analyse data. Multivariate analysis estimates the many factors and their interaction that probably contribute to an observed result. Therefore, research in medicine focuses on the median subject, limiting variability by inclusion and exclusion criteria, and on the study of one factor in an RCT, assuming that variability of all other factors will be taken care of by randomisation.

Clinical medicine deals with individuals and their cultural, geographic, social, economic, racial, and other variabilities. Clinical medicine diagnoses and treats all individuals, including those at the extremes of the Gaussian distribution, including multimorbidity in all age groups within the entire human population.

Research and clinical medicine are deductive and inductive, respectively. Research investigates for each disease, the time course of symptoms and clinical, imaging and laboratory findings. Each aspect is a test, defined by its sensitivity and specificity, from which the clinician calculates the predictive values or the probability that an individual with a positive test has the disease and the risk that a negative test misses the disease. However, for a patient with pelvic pain, for example, the clinician has to consider the probability of each of the many causes of pelvic pain, including rare diseases using History AND symptoms AND clinical exam AND imaging AND all other tests. This explains the gap between the scientific discussion of which sensitivity/specificities/predictive value is good enough versus the clinical considerations of whether a test, when used sequentially, changes our management and what is the risk of harming with a screening test because of false positives and false negatives (Gøtzsche and Jørgensen, 2013; Harris et al., 2014).

Clinical medicine is about the individual person, using the statistical analysis of research data to estimate the indications and risks of alternative diagnoses and treatments.

## Conclusions

Traditional frequentist statistics in medicine are based on normal distributions of variables permitting the calculation of the extremeness or percentile of each value. Similarly, the null hypothesis that the results of a correctly performed trial are not different, is used to calculate the probability that observed difference can be caused by chance, expressed as the p-value. Unfortunately, this p-value is often misunderstood in medicine as indicating that the hypothesis is true, known as the p-value fallacy. The many far-reaching consequences for the medical literature are beyond this manuscript, except that ‘significant’ is nearly meaningless since arbitrarily defined. It is also important that hypothesis testing should be limited to the hypothesis underlying sampling, raising the problems of sub-group analysis and multiple testing.

Bayesian statistics uses new data to update knowledge and to estimate the probability that the null hypothesis or the prediction of an observed difference is true. New research information is used to update our actual understanding. The personal clinical experience is the progressive management update after each new diagnosis, treatment and outcome. Personal clinical experience has low value because of the many potential biases. However, if the personal experiences of many are similar, this collective experience is probably the best we have to judge all aspects of management in the entire population.

Traditional and Bayesian statistics are related. For example, a 50% probability that a drug might be effective (= we do not know) changes into 70% or 80% when an RCT finds a p-value of 0.05 and 0.01, respectively. Low p-values thus increase the probability that the hypothesis will be true (Nuzzo, 2014), although this conclusion cannot be made strictly.

Clinical medicine is different from research since dealing with the entire population and integrating all aspects involved. Therefore, three types of ‘evidence’ need to be considered in medicine: evidence-based, collective-experience-based, and logical deductions when based on circumstantial evidence only.
